# Autophagy in hepatic ischemia–reperfusion injury

**DOI:** 10.1038/s41420-023-01387-0

**Published:** 2023-04-05

**Authors:** Benliang Mao, Wei Yuan, Fan Wu, Yong Yan, Bailin Wang

**Affiliations:** 1 Department of General Surgery, Guangzhou Red Cross Hospital affiliated to Jinan University, Guangzhou, China; 2 College of Clinical Medicine, Guizhou Medical University, Guiyang, China

**Keywords:** Autophagy, Preclinical research

## Abstract

Hepatic ischemia–reperfusion injury (HIRI) is a major complication of liver resection or liver transplantation that can seriously affect patient’s prognosis. There is currently no definitive and effective treatment strategy for HIRI. Autophagy is an intracellular self-digestion pathway initiated to remove damaged organelles and proteins, which maintains cell survival, differentiation, and homeostasis. Recent studies have shown that autophagy is involved in the regulation of HIRI. Numerous drugs and treatments can change the outcome of HIRI by controlling the pathways of autophagy. This review mainly discusses the occurrence and development of autophagy, the selection of experimental models for HIRI, and the specific regulatory pathways of autophagy in HIRI. Autophagy has considerable potential in the treatment of HIRI.

## Facts

- HIRI, which begins with energy metabolism problems, is inseparable from autophagy.
- In the vast majority of cases, activated autophagy has an anti-HIRI effect.
- Autophagy regulates HIRI through numerous pathways, which makes autophagy a powerful potential for treating HIRI.

## Open questions

- Designing a co-culture cell model of hepatocytes and liver inflammatory cells seems to be more conducive to verify the regulatory relationship between autophagy and HIRI.
- Proven and reliable animal experiments on autophagy and HIRI are needed to better translate these results to clinical studies.

## Introduction

The liver receives a dual blood supply, which makes it more susceptible to ischemia and hypoxia [1, 2]. Hepatic ischemia–reperfusion injury (HIRI) is mainly caused by liver surgery, such as partial hepatectomy and liver transplantation, and is an important risk factor leading to poor postoperative outcomes [3]. The occurrence of HIRI follows the two phases of ischemia and reperfusion. During the initial phase, sudden blood-flow interruption exposes hepatocytes to oxygen deprivation, ATP depletion, and enhanced utilization of hepatic glycogen. During the second phase, liver blood flow and oxygen levels are reestablished, and a series of injurious factors such as proinflammatory cytokines, reactive oxygen species (ROS), and later the aseptic inflammatory response emerge, thereby further aggravating hepatocellular damage [4–6]. HIRI is more likely to occur and is more severe when the liver transitions to fatty liver, alcoholic liver, aging liver, and cirrhosis [7, 8]. Although numerous mechanistic studies on HIRI have been conducted, there are currently no definite and effective interventions and treatments [9, 10].

Macroautophagy (referred to as autophagy hereafter) is a process of engulfing the cytoplasmic proteins or organelles and enveloping them into vesicles. After fusing with lysosomes, vesicles form autolysosomes, ultimately degrading the encapsulated contents, thereby realizing the metabolic needs of cells and the renewal of certain organelles [2, 11]. Autophagy occurs both in physiological and pathological processes, and common pathological induction factors include nutrient deficiency, abnormal energy metabolism, ischemia, hypoxia, and pathogen infection [12]. Cells normally maintain basal autophagic activity, unless there are induction factors, such as those mentioned above. Because they are both closely related to the induction of nutrient deficiency, there is also a tight link between HIRI and autophagy. As we will detail in this review, this relation is complex. Namely, autophagy can dramatically regulate multiple aspects of HIRI, from energy metabolism to release of ROS, oxidative stress, mitochondrial dysfunction, and sterile cascade inflammatory response, whereas autophagy is also subject to various regulations by HIRI.

### Outline of the autophagy process

**Fig. 1:**
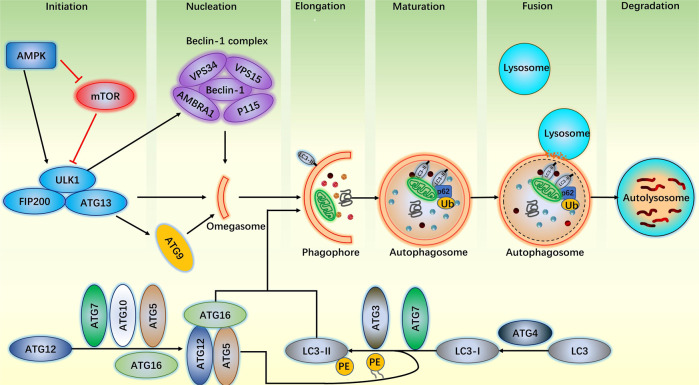
Steps and machinery of autophagy. Stress signals such as hunger, hypoxia, shock, oxidative stress, and inflammatory responses activate AMPK and inhibit mTOR. As their common target, the ULK1 complex triggers the nucleation of phagophore by phosphorylating the Beclin-1 complex and activating ATG9, recruiting membrane sources to form the initial membrane skeleton—omegasome. The ATG12-ATG5-ATG16 complex enhances the conjugation of LC3-II to PE, thereby assembling on the forming phagophore to which autophagic targets are recruited. Targets, which can be labeled by ubiquitin chains, as well as cargo receptors, recruit membrane components to elongate the membrane structure and cargo (damaged organelle, protein aggregate) so as to promote maturation. Cargo needs to be decorated with delicious labels (most prominently ubiquitin (Ub) chains) so that autophagic receptors (p62) recognize it and that it can be swallowed by autophagosome. After lysosomal fusion, the contents of the autolysosome are degraded, and nutrients are recycled by the cell. Abbreviations: ULK1 Unc-51-like kinase 1; ATG autophagy-related protein; FIP200 RB1-inducible coiled-coil protein 1; VPS vacuolar protein sorting; AMBRA1 autophagy and Beclin-1 regulator 1; p115 vesicular transport factor; LC3 microtubule-associated protein light chain 3; PE phosphatidylethanolamine; p62 SQSTM1 gene and sequestosome-1; Ub ubiquitin.

Autophagy is regulated by autophagy-related (ATG) proteins and relies on lysosomal assistance to reclaim and reuptake nutrients from the cytoplasm under conditions of metabolic stress [13]. Autophagy mainly includes six steps: initiation, nucleation, elongation, maturation, fusion, and degradation (Fig. 1).

#### Initiation

Typically, mammalian target of rapamycin (mTOR) is activated and inhibits the initiation of autophagy through direct phosphorylation of the ULK1-ATG13-FIP200 complex (Unc-51-like kinase 1, ATG13, RB1-inducible coiled-coil protein 1) during nutrient abundance [14, 15]. However, when subjected to stress signals such as hunger, hypoxia, shock, oxidative stress, and inflammatory responses, mTOR activity decreases and its inhibition of autophagy is relieved. In addition, adenosine 5′-monophosphate–activated protein kinase (AMPK) can also trigger autophagy directly or indirectly [16]. AMPK is an upstream member of the mTOR pathway. Unlike mTOR, AMPK actively mobilizes autophagy by both phosphorylating the ULK1 complex and repressing mTOR during periods of nutrient deficiency [17].

#### Nucleation

Following autophagy initiation, the activated ULK1 complex promotes nucleation through two pathways. On the one hand, the ULK1 complex phosphorylates the Beclin-1 complex, binds to it, and then locates it to the rough endoplasmic reticulum (ER). On the other hand, the ULK1 complex activates ATG9, recruiting membrane sources to reach the ER. The two pathways work together to promote the formation of double membranes; that is, of omegasome [18, 19].

#### Elongation

Similar to paved railways, the nuclearized membrane skeleton needs to be continuously extended to form a complete autophagosome structure. The process is controlled by two ubiquitin-like systems, including ATG12-ATG5-ATG16 and LC3-PE [19]. Successively transmitted by the ubiquitinated E1-like enzyme ATG7 and ubiquitinated E2-like enzyme ATG10, ATG12 binds to ATG5 and ATG16 to form the ATG12-ATG5-ATG16 complex [20]. In the LC3-PE ubiquitin system, with cleavage at the C terminus by ATG4 proteases, microtubule-associated protein light chain 3 (LC3) proteins expose a terminal glycine residue, and then become LC3-I. The E1-like enzyme ATG7, the E2-like enzyme ATG3, and the E3-like ligase ATG12–ATG5-ATG16 complex transfer LC3-I (cytosolic) to phosphatidylethanolamine (PE), forming a LC3-II-PE complex [11, 13]. LC3-II is anchored in membranes by conjugation to PE and promotes the elongation of isolation membrane by recruiting membrane components.

#### Maturation

The isolation membrane encloses to form autophagosomes. The ATG12-ATG5-ATG16 complex sheds from the outer membrane of the autophagosome, while the LC3-II anchors to the double membrane. In this process, LC3-II plays an important role together with the P62 protein [21]. P62 can promote the packaging of ubiquitinated substrates through self-oligomerization and transport the packaged substrates to LC3-II on the autophagic membrane. P62 interacts with LC3 by its LC3-interacting region (LIR) to form a complex, which is eventually degraded together as an autophagy-specific substrate in autolysosome. Therefore, the dysfunction of autophagy leads to a significant aggregation of p62 [22, 23].

#### Fusion

Ordinarily, autophagosomes first fuse with endosomes to form amphisomes, and then fuse with the outer membrane of lysosomes to form autolysosomes [11]. This fusion causes a single-membrane autophagic body to slide into the lysosomal cavity for better hydrolysis of its contents [13].

#### Degradation

After fusion, acid hydrolases in lysosomes digest autophagic cargo, and the salvaged nutrients are released back into the cytoplasm for reuse by the cells [24, 25].

### Key autophagic factors

The progress of the autophagy pathway requires the orderly coordination of various autophagy-related factors. These autophagy-related factors include, but are not limited to, two pathways (mTOR and AMPK), three complexes (ULK1 complex, Beclin-1 complex, and ATG16 complex), two ubiquitination systems, and several cargo transporters and autophagic receptors (LC3 and p62).

#### mTOR, AMPK, and ULK1 complex

Mammalian target of rapamycin (mTOR), a protein kinase, exists as two complexes: mTOR complex 1 (mTORC1) and mTOR complex 2 (mTORC2) [26, 27]. In this section, we mainly discuss mTORC1, which functions in autophagy initiation with an inhibition role by phosphorylating the ULK1 complex in nutrient-sufficient conditions [28]. Thus, a factor or protein located upstream of mTOR can inhibit autophagy by activating mTOR or induce autophagy by inhibiting mTOR. AMPK is this protein. AMPK can inhibit mTOR and enter the mTOR pathway. When the glucose level is low or the ratio of AMP to ATP is elevated, AMPK is activated and initiates autophagy by phosphorylation of the ULK1 complex [29]. ULK1 complex (ULK1, ATG13, and FIP200) is located in the confluence of the mTOR and AMPK pathways, initiating autophagy by phosphorylated autophagy components.

#### ATG9 and Beclin-1 complex

ATG9, a six-transmembrane protein, is responsible for providing lipid bilayers to form the autophagosome precursors. Furthermore, ATG9 is an important vehicle between the autophagosome membrane and the peripheral vesicle pools, transporting the membrane components to the membrane skeleton for assembly by shuttling back and forth between PAS and vesicle pools [30, 31]. Beclin-1 complex, consisting of the core components Beclin-1, VPS15, and VPS34, constitutes a molecular platform for the regulation of autophagosome formation and maturation [32]. However, the combination of Beclin-1 to Bcl-2 generally results in autophagy in an inhibited state. Unbinding of Bcl-2 from Beclin-1 may be an essential requirement for autophagy upregulation [33, 34].

#### ATG16 complex, LC3, and p62

The ATG16 complex and LC3 are generated by two ubiquitin‐like conjugation systems. For the ATG16 complex, following successive transmissions through ATG7 and ATG10, ATG12 is conjugated to the lysine side chain of ATG5 and further to Atg16 [35, 36]. As for LC3, pro-LC3 is first cleaved by ATG4 (a cysteine protease) to expose a C‐terminal glycine, videlicet LC3-I. LC3-I is a substrate in E1‐like ATG7, E2‐like ATG3, and E3‐like ATG12‐ATG12‐ATG16 complex after sequential ubiquitination modification, scilicet LC3-II, where its C-terminal glycine carboxyl and PE are bound by amide bonds [37]. Finally, ATG4 removes the conjugate link of LC3-PE from the outer membrane of the autophagosomes to promote the maturation of the autophagosomes [38]. The remaining LC3-II interacts with the autophagy receptors to drag the ubiquitinated cargo protein into the autophagosome for easy decomposition. P62 is a member of autophagy receptors families. Functioning in the recognition and encapsulation of degradation substrates in the autophagosome, p62 ultimately decreases with the increase of autophagy, serving as a feedback regulator of the process [39, 40].

### Experimental studies of autophagy in HIRI

It is important to understand the current commonly used methods of liver blood flow blocking in liver surgery. (i) The Pringle maneuver is divided into intermittent liver blood flow blocking and continuous liver blood blocking [41]. Intermittent blocking is commonly used to block the whole liver blood inflow (including the hepatic artery, the portal vein, and even the bile duct), and is suitable for various types of hepatectomy. At present, it is believed that the safe time limit of intermittent blockade is 15 to 20 min, followed by a 5-minute-long revocatory restriction and re-blockade. This process can be repeated multiple times within 120 min. (ii) Whole hepatic vascular exclusion (WHVE) blocks the whole hepatic blood inflow and outflow for 20 to 30 min (including the subdiaphragmatic aorta, hepatic duodenal ligament, subhepatic vein, and vena cava) [42]. (iii) With a better understanding of the liver’s anatomy, hemi-hepatic vascular exclusion and selective hepatic vascular exclusion have also emerged, which can better protect the postoperative liver function [43]. There are also other methods of hepatic blood flow blockade available clinically. For patients with different liver functions, the ischemia time and reperfusion time also change relative to the standard. For example, patients with alcoholic liver, fatty liver, and cirrhosis suffer ischemia for a shorter duration during liver resection or liver transplantation.

#### Experimental subjects and models

Current experimental studies on autophagy and HIRI remain focused on preclinical research. Therefore, isolated hepatocytes (or purchased cell lines) and animals are the main experimental objects. Hepatocytes are used for anoxia (A)/reoxygenation (R) models or to construct gene knockout/overexpression models. Unlike hepatic ischemia (I)/reperfusion (R) models in animals, an A/R model of liver cells facilitates the regulation of the time of cellular hypoxia and reoxygenation, and allows for better management of nutrient concentrations in the medium. However, the occurrence of HIRI is not only based on liver parenchymal cells, but other cells such as hepatic macrophages, hepatic endothelial cells, and neutrophils are also involved in this process. In the fight against HIRI, different kinds of cells play different roles and take on different responsibilities to form a holistic team. Therefore, it is inappropriate to model only the A/R damage of liver parenchymal cells to simulate ischemia–reperfusion damage that occurs throughout the liver. Hence, it is necessary to also establish an animal model of HIRI.

Rats or mice are the most commonly used species for animal HIRI models. Only a small percentage of investigators selected patients as study subjects. To some extent, this shows that the current research on the mechanism of HIRI is not sufficiently developed, and there is no specific drug or mature treatment strategy. Some authors [39] have also used pigs as experimental subjects to study the relationship between autophagy and HIRI in laparoscopic hepatic resection.

For in vitro cell experiments, most researchers set the hypoxia time at 0 to 24 h, and the reoxygenation time ranges from 0 to 48 h. For in vivo experiments, most researchers set ischemia times at 30, 45, 60, and 90 min, with the choice of 60 min being the most common, and reoxygenation time ranges from 0 to 48 h, with the choices of 6, 12, and 24 h being the most common. Ordinarily, after the liver suffers ischemic/reperfusion (I/R) damage, its transaminases begin to decline after peaking at 6 to 12 h of reperfusion [44–47], which indicates that liver injury is most severe after 6 to 12 h of reperfusion. Moreover, the expression of autophagy-related proteins and autophagic fluxes also peak after 6 to 12 h of reperfusion [5, 48–50].

### Which pathways are involved in the effects of autophagy on HIRI?

Over the past decade, numerous studies have confirmed an inextricable link between autophagy and HIRI. Autophagy plays an important regulatory role in energy metabolism, oxidative stress, and inflammatory response in HIRI. Some drugs and treatment strategies with the potential to improve HIRI have been initially explored (Table 1).

**Table. 1:** Summary of the studies of autophagy on HIRI in different experimental models.

Pathway	Therapeutic strategy	Effect of autophagy	Autophagic inhibitors	Refs.
AMPK/mTOR pathway	Everolimus Alda-1 Interleukin 37 (IL37) Melatonin, trimetazidine Isoflurane-Preconditioning	protective	Bafilomycin A1 3-methyladenine (3-MA) Compound C (CC)	[48, 53, 133–135]
PINK1/Parkin pathway	Augmenter of liver- regeneration (ALR) Dj-1 knockout MSCs Pterostilbene	protective	3-MA Chloroquine (CQ)	[136–139]
NLRP3 inflammasome pathway	25-Hydroxycholesterol Knockout of TRPM2 ATP6V0D2	protective	3-MA Rapamycin	[4, 47, 140]
MAPK pathway	Cisplatin 7,8-dihydroxycoumarin Oleanolic acid (OA) Beraprost (BPS)	protective detrimental	CQ	[71, 79, 141, 142]
Sirt1/FoxO3a pathway	Berberine (BBR) 2-Methoxyestradiol HO-1 Nobiletin	protective	Sirt1 inhibitor (EX527) CQ SIRT1 inhibitor (sirtinol)	[7, 94, 98, 99]
PI3K/Akt pathway	Shikonin Rimonabant Suberoylanilide-hydroxamic acid	detrimental protective	CQ	[106, 107, 111]
ER stress	Vitamin D receptor	protective	CQ	[112]
Nrf2/HO-1 pathway	CDDO-imidazole Baicalein Ischemic- Preconditioning Hemin	protective	3-MA CQ Tin protoporphyrin IX (SnPP) Wortmannin Znpp	[115, 117, 119, 120]
miR-20b-5p/ATG7 axis	lncRNA HOTAIR	protective		[124]

#### AMPK/mTOR pathway

**Fig. 2:**
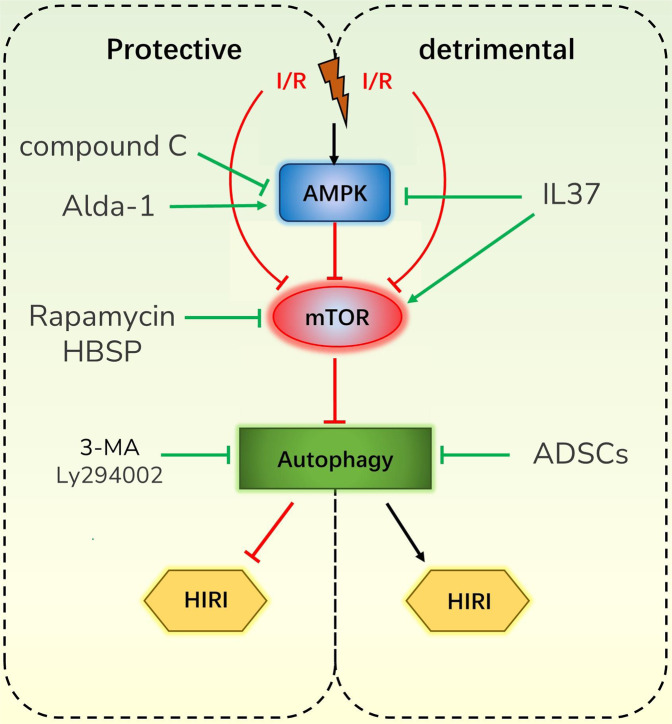
Effects of autophagy on HIRI via the AMPK/mTOR pathway. Abbreviations: IL37 interleukin 37; 3-MA 3-methyladenine; HBSP Helix B surface peptide; Ly294002 Akt inhibitor.

There is no doubt that the AMPK/mTOR pathway is the most important and currently the most well-studied pathway in autophagy. As the pathway closely related to nutrient/energy metabolism, it must also be inextricably linked to the occurrence and development of HIRI. An increasing number of studies have indeed confirmed this expectation (Fig. 2). Moreover, most of the other mechanisms associated with autophagy and HIRI are based on the AMPK/mTOR pathway.

Ge et al. [39] found that when HIRI occurs, the expression and activity of mTOR decrease, and autophagy is induced. In their study, autophagy acted as a bidirectional regulator in the development of HIRI with a protective role in early stages and with a detrimental role during prolonged ischemia. Furthermore, adipose-derived stem cells (ADSCs) can reduce HIRI by inhibiting autophagy. Another research [48] also supported the view that excessive autophagy is harmful. Furthermore, interleukin 37 (IL37) ameliorates HIRI by inhibiting excessive autophagy through inactivating AMPK and ULK1 phosphorylation and facilitating mTOR phosphorylation. Nevertheless, the vast majority of researchers hold the opposite view, and they have experimentally demonstrated that autophagy mainly plays a role in improving HIRI. Liu et al. [51] revealed that spermidine activates autophagy through the AMPK/mTOR/ULK1 pathway, which in turn ameliorates inflammation response and alleviates apoptosis after HIRI in mice. However, an AMPK inhibitor (compound C, CC) inhibits autophagy and weakens the therapeutic effect of spermidine. Correspondingly, in Tan’s study [52], inhibition of mTOR enhances autophagy and strengthens Helix B surface peptide (HBSP)–mediated protection against HIRI. Moreover, the protective effects of HBSP are abolished by an autophagy inhibitor (3-methyladenine, 3MA) and an Akt inhibitor (Ly294002), but enhanced by an mTOR inhibitor (rapamycin). In addition, Li et al. [53] confirmed that Alda-1 can play a hepatoprotective role by phosphorylating AMPK to activate autophagy, and that both 3MA and CC can blunt these protective effects.

#### PINK1/Parkin and NLRP3 inflammasome pathway

The elimination of damaged mitochondria requires a kind of selective autophagy called mitophagy. Its initiation and occurrence are dependent on the PINK1/Parkin pathway [54]. Pink1 (phosphatase and tensin homolog-induced putative kinase 1) degrades in healthy mitochondria to prevent Parkin from being transported to mitochondria [55, 56]. However, when the mitochondria are damaged, PINK1 aggregates into the outer membrane of the mitochondria and phosphorylates ubiquitin and Parkin (an E3 ubiquitin ligase encoded by the gene Park2) to activate Parkin’s E3 enzyme activity and recruit Parkin to the mitochondria [57, 58]. Parkin, located on the mitochondria, then ubiquitinates the outer membrane protein of the mitochondria, making the damaged mitochondria more easily recognized by autophagic receptors and further transported to the autophagosomes for degradation [59]. When the liver suffers I/R damage, a large number of damaged mitochondria are produced. The dysfunctional mitochondria then release large amounts of harmful mitochondrial ROS (mtROS) and mitochondrial DNA (mtDNA) [60, 61]. On the one hand, these mtROS and mtDNA activate the downstream NLRP3 (nucleotide-binding domain leucine-rich repeat-containing family pyrin domain containing 3) inflammatory pathway, resulting in the release of large amounts of inflammatory cytokines to induce inflammatory damage to the liver. On the other hand, mtROS and mtDNA in turn activate the PINK1/Parkin pathway, thereby inducing mitophagy to inhibit the NLRP3 inflammatory pathway and reduce inflammatory damage [62–64].

Some examples illustrate the specific relationship between Pink1/Parkin pathway–induced mitophagy and the NLRP3 inflammasome pathway in HIRI. For instance, Xu et al. found that overexpressed PINK1 induced mitophagy through the PINK1/Parkin pathway, which inhibited the NLRP3 inflammatory pathway and attenuated HIRI. They also showed that 3-MA or chloroquine (CQ) reversed the effects of PINK1 [65]. In addition, Cao et al. found that 25-hydroxycholesterol (25HC) pretreatment–mediated PINK1/Parkin-dependent mitophagy reduced HIRI by inhibiting NLRP3 inflammation. Correspondingly, 3-MA counteracted the protective effect of 25HC [4].

#### MAPK signaling pathway

The MAPK (mitogen-activated protein kinase) pathway is an important signal process that conducts extracellular signal into the cells and regulates cellular activity through a three-kinase cascade. Extracellular signals (such as stress, growth factors, inflammatory cytokines) enter the cells and successively activate MKKK (MAP kinase kinase kinase kinase), MKK (MAP kinase kinase kinase), and MAPK; then, the activated MAPK further regulates cellular growth, differentiation, apoptosis, autophagy, and death. MAPK has four subgroups, including ERK (extracellular signal-regulated kinase), JNK (c-Jun N-terminal kinase), p38, and ERK5. Therefore, the MAPK pathway is divided into four different pathways due to its subgroups, including the ERK/MAPK pathway (i.e., the classic MAPK pathway), the JNK/MAPK pathway, the p38/MAPK pathway, and the ERK5/MAPK pathway [66–70]. In view of the fact that the occurrence, development, and results of the JNK/MAPK pathway and the p38/MAPK pathway are usually quite similar, the two pathways are collectively referred to as the p38/JNK/MAPK pathway. Moreover, joint research on the ERK5/MAPK pathway has not been reported in autophagy and HIRI, so this article focuses on the first three MAPK pathways.

In a mice HIRI experiment, it was found that I/R activated the deleterious p38 and JNK at 1 h after reperfusion, and also activated the beneficial ERK at 3 h after reperfusion. In contrast, cisplatin pretreatment decreased the activation of p38 and JNK, and caused ERK activation to peak at an earlier time point. This hepatoprotective effects of cisplatin are related to the regulation of JNK on autophagy [71]. Wang et al. found that tanshinone IIA (TanIIA) pretreatment mitigated HIRI by activating autophagy through the MEK (subgroup of MKK)/ERK/mTOR pathways [46], which confirms that ERK is also closely related to autophagy and plays a protective role in HIRI. As for the p38/MAPK pathway, remote ischemic preconditioning (RIPC) upregulated the expression of heme oxygenase-1 (HO-1), activated this pathway to initiate autophagy and inhibit apoptosis, and ultimately protected hepatocytes from HIRI [72].

Although ERK, JNK, and p38 are all members of the MAPK family, the ERK/MAPK pathway greatly differentiates from the JNK/p38/MAPK pathway in response to the regulation of autophagy and apoptosis. Moreover, the MAPK pathway regulates autophagy by controlling the state of Beclin-1. In resting cells, Beclin-1 and Bcl-2 bind to maintain basal levels of autophagy. Under autophagy-induced conditions, Bcl-2 dissociates from Beclin-1, resulting in an increase in autophagy levels [73–75]. In HIRI, the MAPK cascade in the cytoplasm is directly activated sequentially after being stimulated by extracellular I/R signaling, but I/R also causes cells to release ROS and inflammatory cytokines, which also promotes the activation of MAPK. Activation of the ERK/MAPK pathway promotes the dissociation of Bcl-2 from Beclin-1, thereby promoting autophagy and inhibiting apoptosis. In contrast, activation of the JNK/p38/MAPK pathway tends to inhibit the dissociation of Bcl-2 from Beclin-1, thereby weakening autophagy and promoting apoptosis [76–78].

However, research on the MAPK pathway in autophagy, apoptosis, and HIRI remains controversial. Some researchers have reported that the p38/JNK/MAPK pathway can be beneficial in activating autophagy and thus aggravating I/R damage to the liver [79, 80]. It has also been suggested that the activation of the JNK/MAPK pathway leads to rapid dissociation of Bcl-2 from Beclin-1, thereby promoting autophagy and alleviating HIRI, but ERK and p38 are not involved in autophagy regulation [81, 82]. Interestingly, another study showed that tri-iodothyronine (T3) preconditioning upregulated ERK phosphorylation to increase autophagy levels, thereby weakening HIRI, while there were no significant changes in the phosphorylation levels of p38 and JNK [83]. This diversity is generated not only from the variability of Beclin-1 and Bcl-2 phosphorylation sites, but also from the localization changes of Beclin-1 and Bcl-2 in cells. These changes ultimately determine whether the cells are headed for survival or death.

#### Sirt1/FoxO3α signaling pathway

Sirtuin 1 (Sirt1) is the best characterized and well-studied protein in the human sirtuin family, a highly conserved NAD^+^-dependent family enzyme that regulates numerous cellular activities, including cellular stress resistance, inflammation, tumorigenesis, and energy metabolism [84–86]. Sirt1 contributes to decreased HIRI by inhibiting mitochondrial damage and oxidative stress, activating autophagic pathway, and enhancing p-ERK, and decreasing p-p38 protein expression [87]. The transcription factor FoxO3α belongs to the FoxO family and is a downstream target of Sirt1 that regulates the expression of genes involved in various biological processes, such as cell death, cell cycle, senescence, and oxidative stress resistance [88, 89]. Sirt1 binds to FoxO3α and deacetylates it, thereby enhancing the regulation of the abovementioned cellular activity processes [90, 91]. An increasing number of studies have shown that the Sirt1/FoxO3α pathway plays an important role in the regulation of autophagy [92, 93].

In general, Sirt1/FoxO3α signaling pathway-mediated autophagy exhibit positive hepatocyte-protective effects [7, 87, 94–97]. Lin and Sheng et al. [98] found that in orthotopic liver transplantation (OLT), expression levels of Sirt1 and FoxO3α increased; moreover, berberine (BBR) administration further increased these expression levels and autophagy, and reduced hepatocyte apoptosis and HIRI. Dusabimana et al. [99] also revealed the protective role of Sirt1/FoxO3α-mediated autophagy in HIRI, and the specific Sirt1 inhibitor EX-527 abolished the protective effect.

#### PI3K/AKT pathway

The phosphoinositide 3-kinase (PI3K)/protein kinase B (AKT) pathway can be activated by multiple types of cellular stimulation or toxic damage. It regulates a variety of basic cellular functions, such as protein synthesis, glycogen synthesis, fatty acid synthesis, apoptosis, and autophagy. First, PI3K catalyzes the production of phosphatidyl inositol-3,4,5-triphosphate (PIP3) on cell membranes. PIP3 then acts as a second messenger, assisting in activating AKT. The activated AKT manipulates key cellular processes such as apoptosis, autophagy, and metabolism by phosphorylating various substrates. The PI3K/AKT is another upstream pathway of mTOR, and it negatively modulates mTOR to promote autophagy [100–105].

There is recent evidence of the important role of PI3K/AKT pathway-mediated autophagy regulation in HIRI. Rezq et al. [106] found that HIRI caused significant activation of the PI3K/Akt/mTOR pathway components and inhibited autophagy. Moreover, the cannabinoid receptor 1 (CB1R) antagonist rimonabant and the autophagy inducer rapamycin both weakened this effect and exerted the protective effects against HIRI. Wang et al. also proposed that inhibiting the AKT/mTOR pathway to activate autophagy can improve OLT-induced HIRI [107]. Although most research has confirmed the hepatoprotective effects of autophagy associated with PI3K/AKT pathway in HIRI [106–110], Liu et al. [111] suggest that the upregulation of autophagy caused by the inhibition of the PI3K/AKT pathway will aggravate HIRI, and related treatments reduce HIRI by activating the PI3K/AKT pathway to inhibit harmful autophagy. The activated PI3K/AKT suppresses subsequent autophagosome formation by inhibiting the dissociation of Bcl-2 from Beclin-1.

#### Other pathways

##### ER stress

Endoplasmic reticulum (ER) stress is initiated when there is an accumulation of unfolded/misfolded proteins and calcium consumption in the ER, to trigger intracellular signal transduction pathways, unfolded protein response (UPR), and maintain intracellular homeostasis [112, 113]. Fang et al. [112] found that activated vitamin D receptor (VDR)–mediated autophagy protects the liver from I/R by inhibiting ER stress.

##### Keap1/Nrf2/HO-1

Nuclear factor erythroid 2-related factor 2 (NRF2) is the primary transcription factor that regulates antioxidant production to maintain cellular redox homeostasis. Under normal circumstances, Nrf2 located in the cytoplasm is degraded by its repressor Kelch-like erythroid-associated protein 1 (Keap1) by ubiquitination and proteasome pathways. Stimulated by certain pathological conditions, Keap1’s obstruction of Nrf2 is lifted, and activated Nrf2 is transported into the nucleus to promote transcription of genes associated with cell protection and antioxidants [114–116]. HO-1 is one of these genes. HO-1–mediated autophagy has been shown to play a positive role in combating HIRI [109, 115, 117–120].

##### microRNA

A variety of microRNAs(miRNAs) have been identified as important regulators of HIRI that either promote or attenuate HIRI [121]. Likewise, autophagy is regulated by miRNAs in multiple pathways [122]. Therefore, some researchers have devoted their attention to the regulation of HIRI by miRNAs through autophagy. Li et al. found that high expression of miR-17 could inhibit Stat3 expression, thereby upregulating autophagy to exacerbate HIRI [123]. Tang et al. found that miR-20b-5p targeted ATG7 and inhibited its expression, attenuated autophagy, and promoted HIRI [124]. Nevertheless, another subfamily of miR-20, miR-20a, can suppress the aberrant expression of genes related to apoptosis and autophagy, protecting against HIRI [125]. The effect of miRNAs to modulate HIRI through autophagy appears to be multifaceted. Different miRNAs induce different autophagic outcomes, which may ultimately lead to aggravation or alleviation of HIRI.

##### Hippo

The Hippo pathway was first discovered in Drosophila and is one of the most conserved pathways across species from prokaryotes to eukaryotes. It appears to have made promising advances in tumor research. In studies of mouse and rat livers, the Hippo pathway is a key regulator of liver size, regeneration, metabolism, and homeostasis [126]. Although few studies have been conducted on the Hippo pathway in the field of organ ischemia-reperfusion injury, it has shown good potential for therapeutic strategies. Liu et al. found that shikonin attenuated myocardial ischemia-reperfusion injury by inhibiting autophagy through the Hippo pathway [127]. Zhou and Chen et al. also reported an important role for the Hippo signaling pathway in HIRI [128, 129]. However, it is unknown whether autophagy regulates HIRI through the Hippo pathway, which provides a possible direction for future studies on the Hippo pathway, autophagy, and HIRI.

This review has only summarized some of the most studied pathways to date, and other mechanisms such as the lncRNA [124], COX-2 [130], CREB-KLF4 axis [131] and Stat3-Atg5 axis [132] may also play an important role in the study of autophagy and HIRI, but more research is required.

## Discussion

**Fig. 3:**
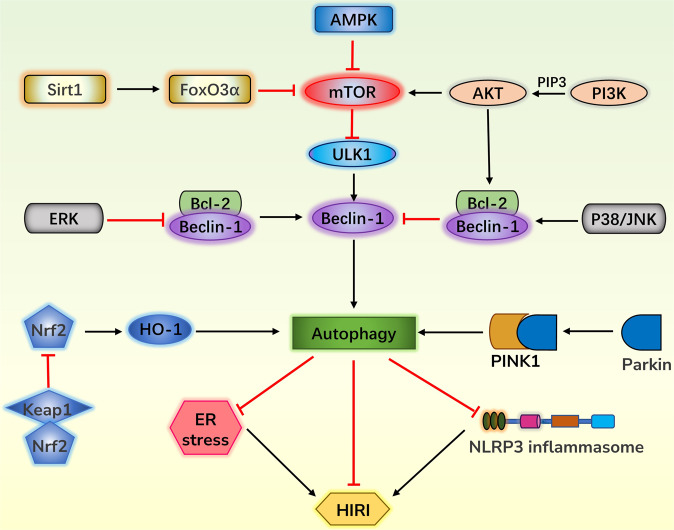
Pathway network for autophagy to affect HIRI. MTOR is the hub of autophagy regulation. The AMPK pathway and Sirt-FoxO3α pathway inhibit mTOR and thus activate autophagy, while the PI3K-AKT pathway activates mTOR and thus hinders autophagy. Beclin-1 is another key protein in the regulation of autophagy. ERK inhibits Bcl-2 and Beclin-1 binding and exerts autophagy activation, while P38 and JNK appear to exhibit opposite effects. The Nrf2/HO-1 pathway and the PINK1/Parkin pathway are also two important pathways that activate autophagy. Finally, activated autophagy can attenuate HIRI either directly or by inhibiting ER stress and suppressing NLRP3 inflammasome. AMPK adenosine 5′-monophosphate-activated protein kinase, mTOR mammalian target of rapamycin, PI3K phosphoinositide 3-kinase, AKT protein kinase B, PIP3 phosphatidyl inositol-3,4,5-triphosphate, Sirt1 Sirtuin 1, FoxO3α transcription factor, ERK extracellular signal-regulated kinase, JNK c-Jun N-terminal kinase, Nrf2 Nuclear factor erythroid 2- related factor 2, Keap1 Kelch-like erythroid-associated protein 1, Pink1, phosphatase and tensin homolog-induced putative kinase 1, Parkin an E3 ubiquitin ligase encoded by the gene Park2, NLRP3, nucleotide-binding domain leucine-rich repeat containing family pyrin domain containing 3.

Autophagy seems to have a star status in the onset and development of HIRI. The pathway network for autophagy to affect HIRI tends to be mature (Fig. 3). With autophagy as a bridge, many drugs and treatments have been reported to have excellent anti-HIRI effects.

A question arises as to whether autophagy has a protective effect or a harmful effect in terms of HIRI. The vast majority of researchers in experimental studies have confirmed that autophagy has a positive hepatoprotective effect, while few have found that autophagy aggravates HIRI. Considering the mechanism of autophagy and the close connection between autophagy and HIRI, autophagy is essentially hepatoprotective when exposed to HIRI. It is worth to carefully analyze the experimental studies that have found that autophagy has liver-damaging effects. First, autophagy is like the body’s inflammatory response; it usually acts as a guardian to protect the human body from external invasion, but once the immune system is impaired, the excessive inflammatory response results in body damage. Under normal circumstances, autophagy only recognizes damaged organelles and misfolded proteins, but excessive autophagy may recognize and degrade normal organelles or proteins. Therefore, excessive autophagy may cause damage to cells. Second, in the studies that have found that autophagy has harmful effects, researchers have mostly only detected changes in the content of autophagy-related proteins and rarely observed the complete process of autophagy. It is not always accurate to assess the autophagy process based solely on autophagy fluxes and the expression of autophagic key proteins. If autophagy remains at a certain stage, such as the maturation or fusion stage, without degradation, then the autophagy process is not completed. At this time, the expression of autophagy-related proteins and autophagic fluxes are deceptive. For such cases, perhaps we can observe the complete process of autophagy by transmission electron microscopy or other means, including from initiation to degradation, and whether there are normal organelles that are engulfed and degraded.

Another point to note is that the application of autophagy inhibitors at different stages of autophagy will result in different manifestations of autophagy. In the initial stage of autophagy, autophagy inhibitors such as 3-MA, wortmannin, and LY294002 can directly hinder the initiation of autophagy, so the expression of autophagy-related proteins such as LC3-II and Beclin-1, and autophagic fluxes are reduced. However, during the fusion phase of autophagy, inhibitors such as Bafilomycin A1 and CQ hinder lysosomal binding to autophagosomes and also hinder the degradation of subsequent autophagic contents, which leads to the reduction of the activity of autophagy and the increased expression of autophagy-related proteins and autophagy flux. This requires correctly selecting autophagy inhibitors in experiments to better detect the occurrence of autophagy.

## Conclusion

To date, extensive research has been conducted to analyze autophagy modulation in liver I/R with the idea to translate these results into clinical practice. Cell and animal experiments have unearthed some powerful therapeutics to improve HIRI by modulating autophagy, but few researchers have made clinical attempts. Hence, well-designed and easily translational animal experiments are needed. Moreover, HIRI is contributed by a variety of cells including hepatocytes, macrophages, endothelial cells, and hepatic stellate cells. However, few studies have examined which cells have the most significant autophagic role in the HIRI process. Maybe co-culture cell models as well as single-cell sequencing would be good solutions.

## Supplementary information

Raw data

## Data Availability

The data that supports the findings of this study are available in the supplementary materials of this article.
