# Correction: Autophagy in hepatic ischemia–reperfusion injury

**DOI:** 10.1038/s41420-023-01436-8

**Published:** 2023-05-05

**Authors:** Benliang Mao, Wei Yuan, Fan Wu, Yong Yan, Bailin Wang

**Affiliations:** 1 Department of General Surgery, Guangzhou Red Cross Hospital affiliated to Jinan University, Guangzhou, China; 2 College of Clinical Medicine, Guizhou Medical University, Guiyang, China

**Keywords:** Autophagy, Preclinical research

Correction to: *Cell Death Discovery* 10.1038/s41420-023-01387-0, published online 05 April 2023

The original version of this article contained an error in the affiliations. Mao Benliang is affiliated with the College of Clinical Medicine, Guizhou Medical University, Guiyang, China and the Department of General Surgery, Guangzhou Red Cross Hospital affiliated to Jinan University, Guangzhou, China. The author also contributed equally to this article. The original article has been corrected.

